# Association of atrial arrhythmias with thrombospondin-1 in patients with acute myocardial infarction

**DOI:** 10.1186/s12872-021-02322-w

**Published:** 2021-10-20

**Authors:** Wenkai Liao, Li Xu, Yuxia Pan, Jie Wei, Peijia Wang, Xinchun Yang, Mulei Chen, Yuanfeng Gao

**Affiliations:** 1 Heart Center and Beijing Key Laboratory of Hypertension, Beijing Chaoyang Hospital, Capital Medical University, Beijing, 100020 China; 2 Department of Cardiology, Chaoyang Hospital, Capital Medical University, 8th Gongtinan Rd, Chaoyang District, Beijing, 100020 China

**Keywords:** Atrial arrhythmias, Atrial fibrillation, Atrial remodeling, Acute myocardial infarction, Thrombospondin-1

## Abstract

**Objectives:**

Atrial remodeling is the main developmental cause of atrial arrhythmias (AA), which may induce atrial fibrillation, atrial flutter, atrial tachycardia, and frequent premature atrial beats in acute myocardial infarction (AMI) patients. Thrombospondin-1 (TSP-1) has been shown to play an important role in inflammatory and fibrotic processes, but its role in atrial arrhythmias is not well described. The purpose of this study was to investigate the role of TSP-1 in AMI patients with atrial arrhythmias.

**Methods:**

A total of 219 patients with AMI who underwent percutaneous coronary intervention and with no previous arrhythmias were included. TSP-1 were analyzed in plasma samples. Patients were classified into 2 groups, namely, with and without AA during the acute phase of MI. Continuous electrocardiographic monitoring was used for AA diagnosis in hospital.

**Results:**

Twenty-four patients developed AA. Patients with AA had higher TSP-1 levels (29.01 ± 25.87 μg/mL vs 18.36 ± 10.89 μg/mL, *p* < 0.001) than those without AA. AA patients also tended to be elderly (65.25 ± 9.98 years vs 57.47 ± 10.78 years, *p* < 0.001), had higher Hs-CRP (39.74 ± 43.50 mg/L vs 12.22 ± 19.25 mg/L, *p* < 0.001) and worse heart function. TSP-1 (OR 1.033; 95% CI 1.003–1.065, *p* = 0.034), Hs-CRP (OR 1.023; 95% CI 1.006–1.041, *p* = 0.008), age (OR 1.067; 95% CI 1.004–1.135, *p* = 0.038) and LVDd (OR 1.142; 95% CI 1.018–1.282, *p* = 0.024) emerged as independent risk factors for AA in AMI patients.

**Conclusion:**

TSP-1 is a potential novel indicator of atrial arrhythmias during AMI.

**Graphical abstract:**

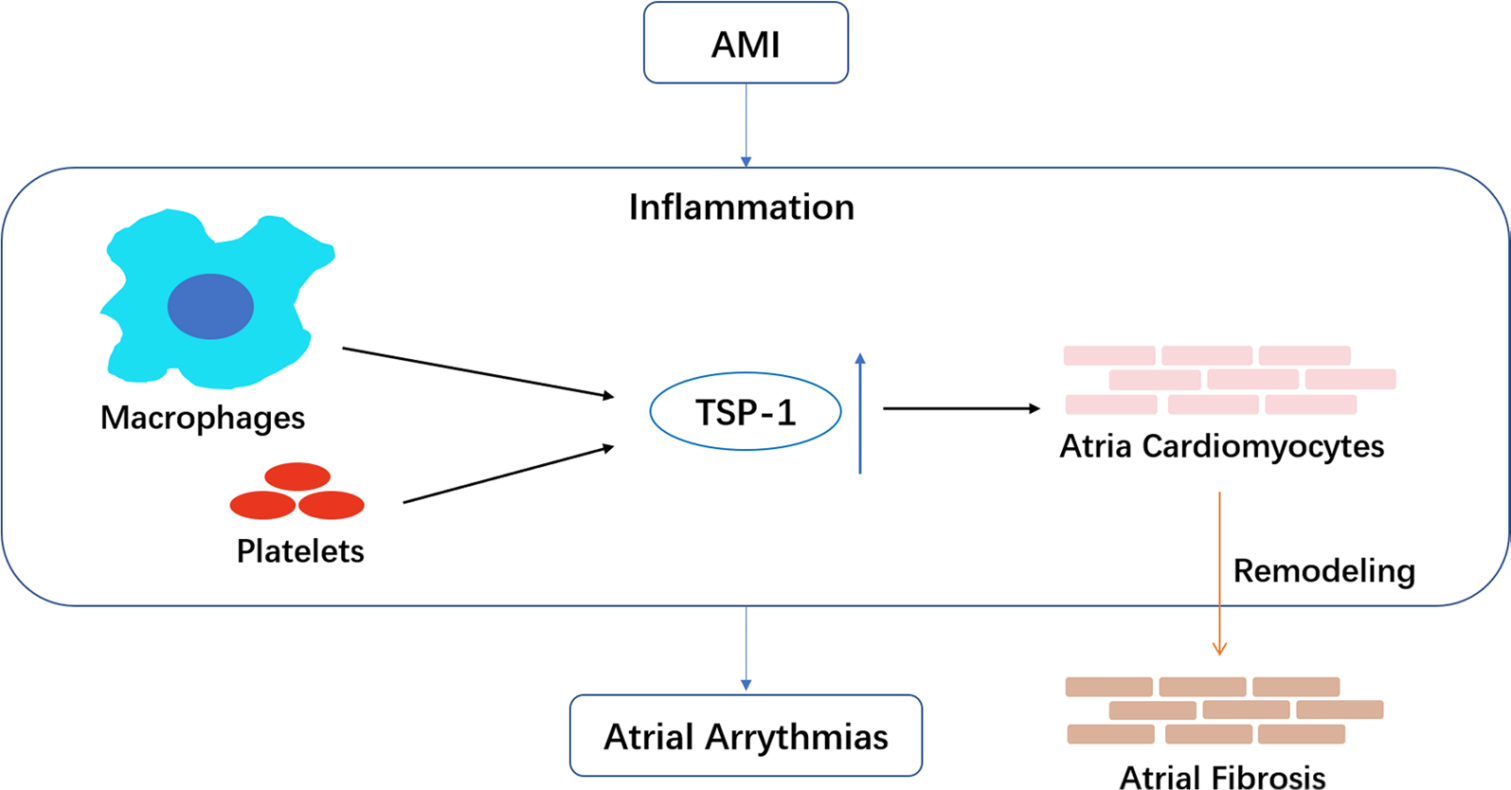

## Introduction

Atrial fibrillation (AF) is one the most frequently encountered arrhythmias. AF affects ~ 33 million worldwide, and both its incidence and associated mortality are increasing. Atrial arrhythmias (AA) are a group of supraventricular arrhythmias including atrial flutter, atrial tachycardia, and premature atrial beats that may eventually develop into AF and therefore requires anticoagulation therapy [1]. Coronary artery disease is one of the main risk factors of AF, along with hypertension, diabetes mellitus, obesity, and age [2].

Acute myocardial infarction (AMI) is one of the most common causes of death worldwide. It is often complicated by supraventricular arrhythmias, the most frequent of which is AF. Patients with AF in AMI have more comorbidities and are at greater risk of reinfarction, strokes, heart failure, and sudden cardiac death [3, 4]. Atrial arrhythmias (AA) like atrial flutter, atrial tachycardia, and premature atrial contractions (PACs) may also develop into AF, increasing the risks mentioned previously [5, 6]. Currently, we only use anticoagulants for AMI patients with new-onset AF for short-term management. Other atrial arrhythmias may have been ignored, despite possibly similar inflammatory mechanisms that eventually lead to atrial remodeling.

**Fig. 1:**
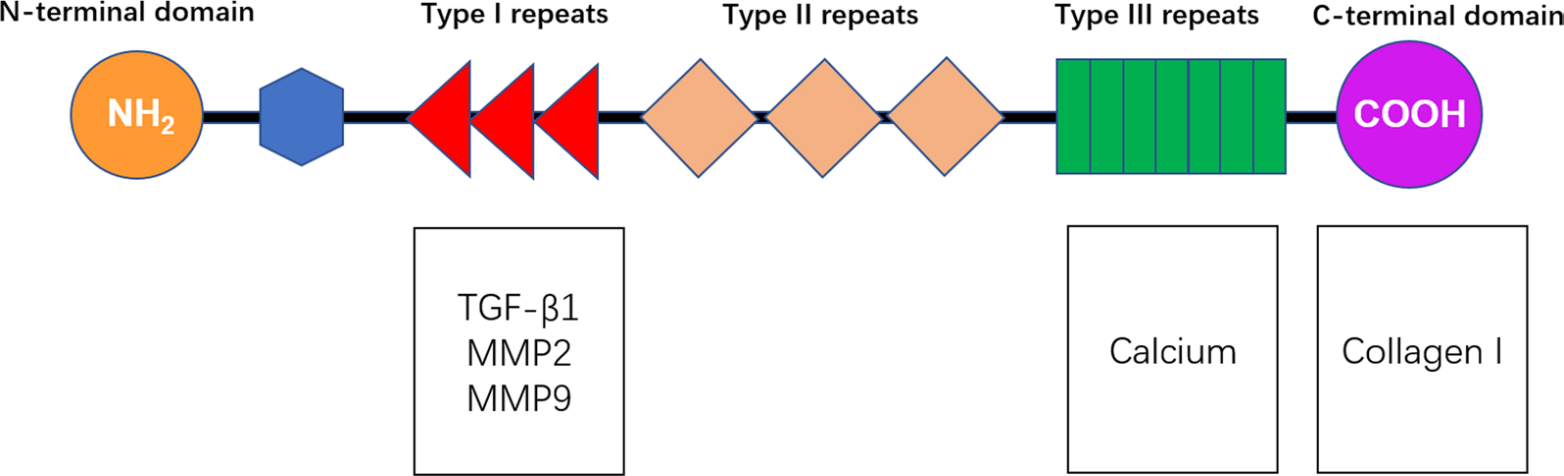
TSP-1 subunit structures and binding partners that regulate atrial ECM

Thrombospondins consist of a family of five atrial extracellular matrix (ECM) member proteins. Of the five, thrombospondin-1 (TSP-1) is the most well described in relation to inflammation and fibrogenesis in many diseases such as liver fibrosis, diabetes, multiple tumors and cardiovascular disorders [7]. TSP-1 is a multi-modular glycoprotein consists of an N-terminal (NH2) domain, three properdin type-I repeats, three epidermal growth factor-like type-II repeats, a calcium-binding type-III repeats, and a C-terminal (COOH) domain that facilitates interactions with other binding partners [8]. TSP-1 is mainly expressed by macrophages and platelets that regulates inflammatory responses through the transforming growth factor-β1 (TGF-β1) pathway [7, 9, 10]. The TGF-β1 pathway initiates inflammation and oxidative stress signaling and is important in ECM regulation and atrial fibrotic remodeling [11]. TSP-1 also interacts with cardiac matrix metalloproteinases (MMPs), collagen I and ionized calcium (Ca^2+^) [12], which are thought to be critical in atrial ECM metabolism [13, 14]. We summarized a figure of TSP-1 binding partners possibly related with atrial ECM remodeling (Fig. 1). AMI acts like a trigger event of an inflammatory response that may induce an increase in TSP-1 and atrial remodeling, causing all kinds of AA, and eventually, AF.

The aim of our study was to compare TSP-1 levels in AMI patients with and without AA as a biomarker for atrial remodeling.

## Materials and methods

### Study population

This study was approved by the Medical Ethics Committees of Beijing Chaoyang Hospital. All patients at the coronary care unit (CCU), Cardiovascular Department, Beijing Chaoyang Hospital who underwent percutaneous coronary intervention (PCI) from 2019 to 2020 were enrolled. A total of 219 patients with AMI and no previous arrhythmias were included. All patients included in this study signed a written consent form. Atrial arrhythmias were defined as the presentation of any kind of atrial electrical abnormalities, including atrial fibrillation, atrial flutter, atrial tachycardia, and premature atrial beats (> 200 beats/day) [15]. Continuous electrocardiographic monitoring (Philips, Eindhoven, Netherlands) was performed to record atrial arrhythmias.

### Laboratory methods

Fasting blood samples were drawn between 6 and 7 a.m. on day 2 after admission. Plasma for determining TSP-1 levels was prepared by centrifugation within 1 h at − 12 °C and 2500 g for 30 min. TSP-1 concentrations were measured by ELISA (Human Thrombospondin-1 ELISA Kit, KE1254, ImmunoWay, Plano, TX, USA). Blood samples for other biochemical characteristics were drawn at the same time and analyzed in a center laboratory of Beijing Chaoyang Hospital.

### Echocardiography

All patients underwent routine transthoracic echocardiography (GE Healthcare Life Sciences, Connecticut, USA) within 3 days after admission. Standard echocardiographic parameters measured included left ventricular end-systolic diameter (LVSd), left ventricular end-diastolic diameter (LVDd), and left atrial antero-posterior diameter (LAAPd). The left ventricle ejection fraction (LVEF) was calculated from apical 2- and 4-chamber views using the Simpson’s biplane method [16].

### Statistical methods

Continuous data were presented as means ± SD, and non-continuous variables were expressed as frequencies. We compared continuous variations using an independent *t*-test and categorical variables using the Pearson χ^2^ test. Univariable analysis was used to identify the risk factors for AA. The potential association between TSP-1 and AA in AMI patients was identified by multivariate logistic regression analysis. SPSS v22.0 (SPSS Inc., Chicago, IL, USA) was used for statistical calculations and illustrations. All tests were two-sided, and *p* < 0.05 was considered statistically significant.

## Results

### Baseline characteristics of AMI patients

The subjects were composed of 186 males (84.9%) and 33 females (15.1%). The youngest patient was 24 years old, whereas the oldest was 79 (mean age 58.32 ± 10.83 years). Of the 219 patients assessed, 24 (11.0%) developed AA, including 16 patients (7.3%) with AF, 1 patient (0.5%) with atrial tachycardia, and 7 patients (3.2%) with frequent PACs > 200/24 h. The type of AF that patients had were all paroxysmal AF and terminated either naturally or with the administration of amiodarone. No patients developed ventricular arrhythmias during the monitoring period. The clinical and biochemical characteristics of AA and non-AA groups are summarized in Table 1.

**Table 1:** Baseline characteristics

	AA (n = 24)	No-AA (n = 195)	AA vs. no-AA, *p*
*Clinical characteristics*			
Age, years	65.25 ± 9.98	57.47 ± 10.78	0.001
Gender, males	19 (79.17)	167 (85.64)	0.403
Weight, kg	69.52 ± 7.64	75.31 ± 9.95	0.007
Heart rate on admission, (per min)	74.79 ± 12.33	77.78 ± 12.00	0.252
SBP on admission, mmHg	122.75 ± 25.20	127.75 ± 19.63	0.256
*AMI type*			
STEMI	14 (58.33)	126 (64.62)	0.545
NSTEMI	10 (41.67)	69 (35.38)	0.545
*Killip classification*			
I	12 (50)	103 (52.82)	0.794
II	7 (29.17)	85 (43.59)	0.177
III	3 (12.5)	5 (2.56)	0.014
IV	2 (8.33)	2 (1.03)	0.012
Patients with history of hypertension	13 (54.17)	102 (52.31)	0.863
Patients with history of diabetes mellitus	9 (37.5)	63 (32.31)	0.609
Patients with history of hyperlipidemia	44 (22.56)	5 (20.83)	0.848
Patients with history of previous myocardial infarction	6 (25)	30 (15.38)	0.230
Patients with history of stroke	3 (12.5)	22 (11.28)	0.859
Family members with premature cardiovascular disease	12 (50)	68 (34.87)	0.084
Use of β-blockers	11 (45.83)	135 (69.23)	0.022
Use of ACEI/ARB	4 (16.67)	82 (42.05)	0.016
*Echocardiography*			
LAAPd, mm	38.46 ± 5.12	37.31 ± 21.94	0.799
LVDd, mm	50.29 ± 4.15	47.83 ± 4.08	0.006
LVSd, mm	36.04 ± 5.95	32.13 ± 5.48	0.001
LVEF, %	50.83 ± 12.62	59.16 ± 10.57	< 0.001
*Biochemical characteristics*			
Hemoglobin, g/L	133.33 ± 16.41	140.76 ± 24.02	0.143
Total cholesterol, mmol/L	4.02 ± 0.94	4.59 ± 1.13	0.018
HDL, mmol/L	0.97 ± 0.29	0.95 ± 0.22	0.615
LDL, mmol/L	2.50 ± 1.09	3.03 ± 0.99	0.016
cTnI, ng/mL	81.41 ± 115.33	92.10 ± 121.38	0.683
BNP, pg/mL	1177.6 ± 1269.00	218.34 ± 348.10	< 0.001
Creatinine, μmol/L	112.45 ± 117.84	70.13 ± 23.31	< 0.001
Hs-CRP, mg/L	39.74 ± 43.50	12.22 ± 19.25	< 0.001
ESR, mm/h	15.29 ± 14.98	16.72 ± 59.95	0.914
TSP-1, μg/mL	29.01 ± 25.87	18.36 ± 10.89	< 0.001

Data are presented as mean ± SD, n (%)

AA, atrial arrhythmias; SBP, systolic blood pressure; AMI, acute myocardial infarction; STEMI, ST-elevation myocardial infarction; NSTEMI, non-STEMI; ACEI, Angiotensin-converting enzyme inhibitors; ARB, Angiotensin II type I receptor blockers; LVEF, left ventricle ejection fraction; LVDd, left ventricle end-diastolic diameter; LVSd, left ventricle end-systolic diameter; LAAPd, left atrium anterior–posterior diameter; HDL, high-density lipoprotein LDL, low-density lipoprotein; cTnI, cardiac troponin I; BNP, brain natriuretic peptide; Hs-CRP, high sensitivity-C reactive protein; ESR, erythrocyte sedimentation rate; TSP-1, thrombospondin-1

Patients with AA tended to be older compared with patients without AA (65.25 ± 9.98 vs 57.47 ± 10.78 years, *p* = 0.001). No significant differences were observed between the two groups regarding gender, heart rate, and systolic blood pressure (SBP) on admission, AMI type, previous hypertension history, diabetes mellitus, hyperlipidemia, myocardial infarction, stroke, and family history of coronary heart disease (*p* > 0.05).

Data showed worse heart functions in patients with AA. Patients with AA had higher Killip levels (Killip III: 12.5% vs 2.56%, *p* = 0.014, Killip IV: 8.33% vs 1.03%, *p* = 0.012), higher brain natriuretic peptide (BNP) levels (1177.67 ± 1269.00 pg/mL vs 218.34 ± 348.10 pg/mL, *p* < 0.001), larger LVDd (50.29 ± 4.15 mm vs 47.83 ± 4.08 mm, *p* = 0.006) and LVSd (36.04 ± 5.95 mm vs 32.13 ± 5.48 mm, *p* = 0.001), and lower LVEF (50.83% ± 12.62% vs 59.16% ± 10.57%, *p* < 0.001). On the other hand, no significant differences were observed in the LAAPd (*p* > 0.05) since all patients had no previous arrhythmias. Patients with AA also used fewer β-blockers (45.83% vs 69.23%, *p* = 0.022) and angiotensin-converting enzyme inhibitors (ACEI)/angiotensin II type I receptor blockers (ARBs) (16.67% vs 42.05%, *p* = 0.016) probably because of worse acute cardiac conditions.

Patients with AA also had lower total cholesterol (4.02 ± 0.94 mmol/L vs 4.59 ± 1.13 mmol/L, *p* = 0.018), lower low-density lipoprotein (LDL, 2.50 ± 1.09 mmol/L vs 3.03 ± 0.99 mmol/L, *p* = 0.016), and lower body weights (69.52 ± 7.64 kg vs 75.31 ± 9.95 kg, *p* = 0.007). No significant difference in high-density lipoprotein (HDL) was observed between the groups (*p* > 0.05).

Patients with AA had higher creatinine levels (112.45 ± 117.84 μmol/L vs 70.13 ± 23.31 μmol/L, *p* < 0.001) than patients without AA, whereas no differences were observed in hemoglobin and cardiac troponin I (cTnI) levels (*p* > 0.05).

The plasma TSP-1 levels (29.01 ± 25.87 μg/mL vs 18.36 ± 10.89 μg/mL, *p* < 0.001) were significantly higher in patients with AA than patients without. High sensitivity-C reactive protein (Hs-CRP, 39.74 ± 43.50 mg/L vs 12.22 ± 19.25 mg/L, *p* < 0.001) was also higher in AA patients. No differences in erythrocyte sedimentation rate (ESR) levels were observed between the groups (*p* > 0.05).

### Multivariate logistic regression reveals TSP-1 as an independent risk factor for newly onset atrial arrhythmias

Univariate analysis for AA risk assessment was performed for the significantly different variables mentioned above. A multivariate logistic regression model was built to find risk factors for AA. As shown in Table 2, TSP-1 (OR 1.042; 95% CI 1.013–1.072, *p* = 0.004), age (OR 1.091; 95% CI 1.033–1.153, *p* = 0.002), Hs-CRP (OR 1.027; 95% CI 1.012–1.043, *p* = 0.001), and LVDd (OR 1.142; 95% CI 1.018–1.282, *p* = 0.024) emerged as independent risk factors for AA after adjusting for other potential risk factors.

**Table 2:** Factors independently associated with newly onset AA in AMI patients

	Univariate			Multivariate		
	OR	95% CI	*p* value	OR	95%CI	*p* value
TSP-1	1.041	1.013–1.069	0.003	1.042	1.013–1.072	0.004
Age	1.083	1.031–1.132	0.001	1.091	1.033–1.153	0.002
Hs-CRP	1.029	1.016–1.042	0.000	1.027	1.012–1.043	0.001
LVDd	1.150	1.038–1.273	0.007	1.142	1.018–1.282	0.024
LVEF	0.937	0.902–0.927	0.001			
BNP	1.001	1.000–1.002	0.001			
Killip level	1.734	0.979–3.072	0.059			
Creatinine	1.014	1.003–1.024	0.008			
Weight	0.931	0.885–0.980	0.006			

TSP-1, Thrombospondin-1; OR, odds ratio; CI, confidence interval; Hs-CRP, high sensitivity-C reactive protein; BNP, brain natriuretic peptide; LVEF, left ventricle ejection fraction; LVDd, left ventricle end-diastolic diameter

## Discussion

AF is becoming more and more prevalent in clinical settings, and yet, the mechanisms are still poorly understood. However, the substrates of AF occurrence are increasingly being examined, improving current knowledge beyond the clinical classification of AF. Aguilar et al. [17] found a model demonstrating that the occurrence of AF is determined by the AF substrate, which in turn is governed by unmodifiable conditions, like genetic influences, aging, and modifiable conditions, like cardiovascular disease and obesity. AF is also determined by the transient substrate, which is produced by a triggering event (like cardiac surgery or an acute illness), inducing a transient increase in inflammation signaling, oxidative stress, and hemodynamic changes. The gradual, time-related progression of the AF substrate eventually reaches the AF threshold, and spontaneous AF occurs. Cumulative trials have also proved atrial arrhythmias like premature atrial contractions, atrial flutter, and atrial tachycardia may convert into AF and thus needs anticoagulation treatment [1, 15, 18, 19]. We hypothesized that the triggering event (AMI in our study) may trigger all kinds of AA with possibly homologous mechanisms. In our trials, the plasma TSP-1 levels (29.01 ± 25.87 μg/mL vs 18.36 ± 10.89 μg/mL, *p* < 0.001) were significantly higher in patients with AA than patients without, which indicates patients with post-AMI AA, other than AF, may also need evaluation for potential risks of stroke and heart failure.

TSP-1 is mainly expressed by macrophages and platelets that may regulate atrial remodeling through multiple pathways, mainly through the TSP-1/TGF-β1 pathway. TSP-1 may bind and activate latent TGF-β, promoting the inflammatory response via recruitment of inflammatory cells and increase myofibroblast differentiation. Besides the TGF-β1 pathway, the C‑terminal domain of TSP‑1 may bind to collagen I directly, regulating fibroblast activities [20]. TSP‑1 may also interact with MMP-2 and MMP-9, possibly inhibiting their activity and regulating collagen homeostasis [21]. Procter et al. [22] also found platelet hyperaggregability, induced by the release of TSP-1 from platelet α-granules, may diminish nitric oxide signaling, thus promoting inflammation. Recently, Zhou et al. [23] reported that microRNA-221 could inhibit latent TGF-β1 activation by targeting TSP-1 to attenuate cardiac fibrosis, which could be a possible upstream treatment of AA.

Previous studies showed conflicting evidence of TSP-1 during AMI. One study reported that TSP-1 could have a protective role in preventing the expansion of healing myocardial infarcts [24], but another stated that low TSP-1 could be associated with major adverse cardiac events in ST-elevation myocardial infarction [25]. Befekadu et al. [26] found TSP-1 rapidly declined after P CI even significantly lower than in healthy individuals. TSP-1 could probably be unrelated to infarct size of heart. In our study, all patients had undergone PCI before blood sample analyses for TSP-1. Our results also showed no correlation between cTnI and TSP-1 (*p* = 0.211).

As a traditional marker of inflammation in AA, Hs-CRP levels have been found significantly higher in patients with AA [27]. However, the substantial mechanism involving Hs-CRP remains unclear. In our study, significant differences in Hs-CRP levels were found between the two groups. We also found that AMI patients with AA were mostly elderly and had cardiac functions and greater levels of creatinine, which were consistent with previous studies [28–30]. Patients with AA in our study needed lesser ACEI/ARBs and β-blockers probably due to limitations of renal and heart functions. We also found lower LDL, which was consistent with a previous study [31], as well as total cholesterol and body weight, in AA patients. Of all the factors that were significantly different between the AA and non-AA groups, only TSP-1, Hs-CRP, and age were found statistically significant by multivariate logistic regression analysis in our predictive model, which suggests a possibly stronger relationship between the incidence of AA and inflammation as well asage.

In our study, TSP-1 worked as a potential novel indicator of atrial arrhythmias during AMI. AMI patients with higher serum TSP-1 levels were more likely to develop AA than the other patients. TSP-1 could also be indicators for AA prediction without AMI. Further studies are needed to explore the relationships between TSP-1 and AA prediction, severity and even relapse after treatment. Lastly, TSP-1 could be possible treatment target for atrial arrythmias.

## Conclusion

In summary, TSP-1 is an important component of the inflammatory response that contributes to atrial remodeling. It may serve as a potential indicator of atrial arrhythmias post AMI, and thus indicating the need for anticoagulation and possible upstream treatments.

## Limitations

Our study had several limitations. Firstly, the sample size of the AA group was relatively small. However, as a hypothesis-generating study, the present study offers valuable insights towards the clarification of the relationship between TSP-1 and new-onset AA post AMI, as well as on the possible substrate of post-AMI AA patients (with preexisting atrial remodeling). Secondly, the prevalence of AA and the long-term outcomes still need further investigation to elucidate the potential use of TSP-1 in risk stratification.

## Data Availability

The datasets generated and/or analyzed during the current study are not publicly available due to the restrictions of human genetics data policy of Beijing Chaoyang Hospital Ethics Committee, but are available from the corresponding author on reasonable request.
